# Supplementary material for: Isolation and identification of hyaluronan-degrading bacteria from Japanese fecal microbiota
Source: PLoS One. 2023 May 17;18(5):e0284517. doi: 10.1371/journal.pone.0284517 (PMC10191264; doi:10.1371/journal.pone.0284517)
Supplement: Attachment — Submitted filename: Cmment for the HA submission.doc [file pone.0284517.s001.doc]

Thanks a lot for the submission. The authors tried to detect Hyaluronan-degrading bacteria in human gut. I have just a couple of comments and suggestions for them.

1 In line 15, you may consider changing subject study participants or donors as used more frequently in the write up. This may be effected through out the write up.
2 In line 18, you may consider restructuring the statement as ELISA cannot select but can be used to confirm the presence the presence or exposure to an entity. Likewise, the statement in line 87
3 You may consider changing the word “absorption” in line 91 to absorbance or optical density.
4 Statement “And then 50 99 μL was sucked up from these each eight wells with an 8-channel pipet…” in lines 98-100 needs restructuring
