# Supplementary material for: Isolation and identification of hyaluronan-degrading bacteria from Japanese fecal microbiota
Source: PLoS One. 2023 May 17;18(5):e0284517. doi: 10.1371/journal.pone.0284517 (PMC10191264; doi:10.1371/journal.pone.0284517)
Supplement: Attachment — Submitted filename: Response to Reviewers.docx [file pone.0284517.s003.docx]

Point-by-point answers to Reviewer’s comments

Thank you for your constructive comments to our manuscript to PLoS ONE. We have revised the manuscript in accordance with your comments as follows:

1. In lines 15, 23, 49, 252, 442 we have changed “feces of healthy Japanese subjects” to “feces of healthy Japanese donors” as you pointed out.

2. In lines 17-18 and 89-90, we have revised the statement of ELISA.

3. In lines 90-91, the word “absorption” was changed to “absorbance”.

4. We have revised the statement about the step of dilution of fecal inoculum in lines 98-101.
